# Role of aggressive locoregional surgery in treatment strategies for ipsilateral supraclavicular lymph node metastasis of breast cancer: a real-world cohort study

**DOI:** 10.3389/fmolb.2023.1248410

**Published:** 2023-10-17

**Authors:** Kexin Feng, Zeyu Xing, Qichen Dai, Han Cheng, Xiang Wang

**Affiliations:** Department of Breast Surgical Oncology, National Cancer Center, National Clinical Research Center for Cancer, Cancer Hospital, Chinese Academy of Medical Sciences and Peking Union Medical College, Beijing, China

**Keywords:** breast cancer, supraclavicular lymph node, supraclavicular lymph node dissection (SLND), survival, prognostic factors

## Abstract

**Background:** Breast cancer patients with synchronous ipsilateral supraclavicular lymph node metastases (ISLNM) have unfavorable prognoses. The role of supraclavicular lymph node dissection (SLND) as a surgical intervention in the treatment of this condition remains controversial. In this study, we aimed to evaluate the prognostic factors associated with breast cancer with ISLNM and to assess the potential impact of aggressive locoregional surgical management on patient outcomes.

**Methods:** We conducted a retrospective analysis of 250 breast cancer patients with ISLNM who were treated with curative intent at our institution between 2000 and 2020. The cohort was stratified into groups based on the extent of axillary surgery. The first group, comprising 185 patients, underwent level I/II axillary dissection. The second group, consisting of 65 patients, underwent aggressive locoregional surgery, including levels I/II/III (infraclavicular) dissection in 37 patients and levels I/II/III + SLND in 28 patients. Our study evaluated overall survival (OS) and disease-free survival (DFS) as primary endpoints, and locoregional recurrence-free survival (LRRFS) and distant metastasis-free survival (DMFS) as secondary endpoints.

**Results:** The median follow-up time among all patients was 5.92 years (1.05–15.36 years). The 5-year OS rate was 71.89%, while the DFS rate, LRRFS rate, and DMFS rates were 59.25%, 66.38%, and 64.98%, respectively. A significant difference in OS, DFS, LRRFS, and DMFS was observed between the second group and the first group (*p* < 0.01). No beneficial impact on recurrence, metastasis, or survival outcomes was observed in the levels I/II/III + SLND group compared to the levels I/II/III dissection group. Multivariate logistic regression analysis revealed that levels I/II/III ± SLND surgery and T stage were associated with OS (*p* = 0.006 and *p* = 0.026), while levels I/II/III ± SLND surgery, ER+/HER2-, and histologic grade were associated with DFS (*p* = 0.032, *p* = 0.001, *p* = 0.032).

**Conclusion:** Breast cancer with ISLNM may be considered a locoregional disease, requiring a combination of systemic and local therapies. Aggressive locoregional surgery has been shown to positively impact recurrence, metastasis, and survival outcomes. This approach may provide improved management of the ISLNM for breast cancer patients.

## 1 Introduction

The occurrence rate of ipsilateral supraclavicular lymph node metastases (ISLNM) in breast cancer patients who do not exhibit any distant metastases varies between 1% and 4% (Chen et al., 2006; Chen et al., 2006). The lymphatic dissemination of axillary breast cancer often adheres to a specific pattern: it initiates in the axillary nodes on the same side as the affected breast, advances to the infraclavicular nodes, and ultimately involves the supraclavicular nodes. It is noteworthy that a significant majority of cases with invasive lobular cancer of the breast with ISLNM are accompanied by metastases in the axillary lymph nodes, with a range of 94%–100%.The presence of ISLNM serves as a noteworthy prognostic indicator, indicating an adverse prognosis. A considerable proportion of these individuals demonstrates the occurrence of distant metastases after a year of identifying isolated tumour cells in regional lymph nodes. Furthermore, the survival rate after 5 years remains disappointingly low, ranging from 5% to 34% (Brito et al., 2001). In the past, the classification of ISLNM in breast cancer was considered as a form of distant metastasis. Consequently, the treatment strategy mostly emphasised palliative measures, whereas local surgical treatments received less attention. Nevertheless, the dynamic and progressive nature of diagnostic and therapeutic developments has introduced a positive outlook. Although the incidence of distant metastases is high in patients with ISLNM, there is a possibility of curative interventions for these individuals. In support of this shift in perspective, the American Joint Committee on Cancer, in its sixth edition TNM staging system (2002), revised the classification of breast cancer patients with isolated tumour cells in regional lymph nodes from stage M1 to clinical N3c (cN3c). The reclassification in question highlights the notion of ISLNM as a localised ailment rather than a disorder characterised by distant metastasis (Singletary et al., 2002). According to the NCCN guidelines (Gradishar et al., 2022), patients with ISLNM should undergo complete or partial breast resection and axillary level I/II lymph node dissection, preceded by preoperative chemotherapy/targeted therapy and followed by postoperative radiotherapy (RT) to the chest wall, supraclavicular, and/or internal mammary lymph nodes. However, it has been reported that the complete response (CR) rate of supraclavicular lymph nodes (SLN) following neoadjuvant chemotherapy (NAC) is only 50% (Zhu et al., 2019), and residual tumor load is a significant concern. Current radiation therapy in the supraclavicular and subclavian regions is limited by normal tissue tolerance and achieving curative agents’ volume is difficult. Simply increasing the radiation dose does not improve patient prognosis but rather increases the occurrence of radiotherapy-related complications.

In addition to primary breast tumor surgery and axillary level I/II dissection, aggressive locoregional surgery, such as axillary level III (infraclavicular) and SLND, may offer a more precise and thorough therapeutic effect than radiotherapy. This approach theoretically reduces local tumor load and prevents the spread of tumor cells through lymph-vessels. Additionally, the surgical removal of the metastatic SLN can provide a more precise assessment of the SLN status following NAC. However, it is crucial to acknowledge that increased radiation doses may heighten the likelihood of problems following radiotherapy (Huang et al., 2007). The existing body of literature lacks a sufficient number of comparative trials that investigate the results of SLND surgery in comparison to neck RT, and the establishment of definite guidelines remains challenging. A comprehensive review of the existing literature reveals a prevalent lack of consensus regarding the optimal localised therapy approach. The objective of this research study was to investigate the possible advantages of intensive locoregional surgical interventions in improving the prognosis for patients with ISLNM and identifying individuals who may benefit from more aggressive therapies. In addition, we initiated an assessment of prognostic factors associated with survival rates in breast cancer patients affected by ISLNM, along with a thorough examination of research focused on SLND. The results of our study have the potential to enhance the existing NCCN guidelines on the management of locally advanced breast cancer by providing additional clinical perspectives.

## 2 Methods and materials

### 2.1 Patients

With approval from the institutional review board of the National Cancer Center/National Clinical Research Center for Cancer/Cancer Hospital, a total of 250 patients with ISLNM and no evidence of distant metastatic disease were identified retrospectively. These patients received intended curative multidisciplinary therapy, which consisted of neoadjuvant systemic medication, surgical resection, and adjuvant radiotherapy (RT). The treatment was administered at our institution over the period from 2000 to 2020.

Complete physical examination and accessory tests were used for preoperative diagnosis, including ultrasonography (US), chest CT, breast magnetic resonance imaging (MRI), and/or positron emission tomography/computed tomography scans (PET/CT). Inclusion criteria were as follows: primary breast tumor confirmed by biopsy, ISLNM confirmed by biopsy or needle cytology, ISLNM defined as cervical lymph node metastasis levels IV and VB, no internal mammary lymph node metastasis or distant metastasis found, no previous history of other malignant tumors, and ability to accept comprehensive treatment, including chemotherapy, targeted therapy, and radiotherapy, without serious organ dysfunction. Patients were included regardless of tumor stage and pathotype.

Patients who had a prior history of breast cancer or other malignancies, as well as those experiencing significant problems in other organs, were excluded based on predetermined criteria. Participants were ineligible for inclusion if they presented with additional distant metastases during the initial treatment, which included surgery or prior chemotherapy. In order to consider the impact on the M stage, we choose to omit patients who exhibited indications of neck lymph node metastases in levels I, II, III, and VI.

### 2.2 Clinicopathological data

The clinical data of the patients were collected, including age at first diagnosis, clinical TNM stage, histology, receptor status, use of chemotherapy, pathological response of the tumor, axillary lymph node, and SLN after NAC, pathologic response, type of surgery and nodal dissection, radiation technique, and initial and boost radiation dose. This information was used to analyze the effectiveness of aggressive locoregional surgical treatment for improving the prognosis of patients with ISLNM.

### 2.3 Treatment

The treatments were administered in accordance with established national criteria and were customised to suit the unique circumstances of each individual patient. After receiving an initial breast cancer diagnosis, participants underwent conventional treatment and underwent routine follow-up, which included physical and imaging exams every three to 6 months following breast surgery. Patients with ISLNM were provided with personalised treatment strategies, which encompassed systemic therapeutic approaches such as chemotherapy, endocrine therapy, and targeted therapy specifically targeting HER2. Additionally, local treatment modalities such as neck RT and/or SLND surgery were employed.

### 2.4 Outcomes and follow up

In our investigation, we assessed recurrences at local, regional, and distant levels. Local recurrences refer to instances where the cancer reappears in either the same breast or the chest wall on the same side. On the other hand, recurrences that occur in the axillary node on the same side or the internal mammary sentinel lymph node (ISLN) are classified as regional recurrences. Tumours that reoccur outside the specified locoregional borders are classified as distant recurrences. The interval between the commencement of systemic therapy to the initial documented relapse was designated as disease-free survival (DFS). This statistic included occurrences of recurrence at the local, regional, or distant level, as well as instances of fatality. The metric known as locoregional recurrence-free survival (LRRFS) was defined as the duration until the initial occurrence of a local or regional recurrence, including recurrences inside the mediastinum or sternum. Overall survival (OS) was measured from the time of surgery to death from any cause or last follow-up. Distant metastasis-free survival (DMFS) was measured to the first occurrence of distant metastasis.

### 2.5 Statistical analysis

The comparison of clinicopathologic categorical factors was conducted using Pearson’s chi-square test. The researchers utilised the Kaplan-Meier method to construct survival curves for factors related to time-to-event. Subsequently, the differences between the curves were evaluated by log-rank testing. A univariate Cox regression analysis was performed to investigate the relationship between clinicopathological characteristics and the likelihood of recurrence. The subsequent multivariate Cox proportional hazards regression analysis considered variables with a *p*-value of less than 0.15 to be relevant and included them. The statistical analyses were conducted using SPSS 22.0 (IBM Corp.) and R 4.1.2 (obtained from https://www.r-project.org/). A significance level of 0.05 was established as the threshold for statistical significance in the bilateral test.

## 3 Results

### 3.1 The clinicopathological characteristics of patients

This analysis incorporated data from a sequential sample of 250 patients. Out of the total sample, 185 patients (74.0%) received sole level I/II axillary dissection, 37 patients (14.8%) underwent dissection of levels I/II/III (infraclavicular), and 28 patients (11.2%) underwent dissection of levels I/II/III in conjunction with supraclavicular lymph node dissection (SLND). The provided information offers valuable insights into the surgical methodologies employed for the management of individuals diagnosed with isolated spinal metastatic lesions. Biopsy tissue test confirmation of the SLN was performed in 249 patients (99.6%). The reason for the 1 patient without a biopsy tissue test was that the result of the PET-CT scan was sufficient for diagnosis. The median age of the patients was 57 years, with a range of 24–75 years. In terms of clinical T stage, 47 patients (18.8%) were classified as T1, 116 patients (46.4%) as T2, 51 patients (20.4%) as T3, and 36 patients (14.4%) as T4. Among the 250 patients, 141 (56.4%) were estrogen receptor (ER) positive and 93 (37.2%) were human epidermal growth factor receptor 2 (HER2) positive. The clinicopathological characteristics of this cohort are summarized in Table 1.

**TABLE 1:** Patient and treatment characteristics.

Variable	All patients (N = 250) (n, %)
Age, years	—
<50	108 (43.2)
≥50	142 (56.8)
Pre-NAC largest SLN size^a^	—
<1 cm	116 (46.4)
≥1 cm	134 (53.6)
Clinical T-stage	—
T1	47 (18.8)
T2	116 (46.4)
T3	51 (20.4)
T4	36 (14.4)
Histology	—
Invasive ductal carcinoma	247 (98.8)
Others	3 (1.2)
Histologic grade	—
Low	60 (24.0)
Median	142 (56.8)
High	48 (19.2)
Receptor Status	
ER+/HER2-	96 (38.4)
ER+/HER2+	45 (18.0)
ER-/HER2+	48 (19.2)
ER-/HER2-	61 (24.4)
Type of primary breast surgery	—
Yes	246 (98.4)
No	4 (1.6)
Type of primary nodal surgery	—
Level I/II	185 (74.0)
Level I/II/III	37 (14.8)
Level I/II/III + SLND	28 (11.2)
Tumor chemotherapy response (ypT0)	—
Yes	63 (25.2)
No	183 (73.2)
NA	4 (1.6)
Nodal pathologic complete response (ypN0)	—
Yes	68 (27.2)
No	178 (71.2)
NA	4 (1.6)
SLN radiographic response to NAC^a^	—
Yes	150 (60.0)
No	100 (40.0)
Involvement of infraclavicular lymph nodes after NAC	—
Yes	26 (10.4)
No	224 (89.6)
Post-NAC largest SLN size^a^	—
<1 cm	224 (89.6)
≥1 cm	26 (10.4)
SLN cumulative dose, Gy	—
<60	172 (68.8)
≥60	56 (22.4)
NA	22 (8.8)
Adjuvant targeted therapy	—
Yes	41 (16.4)
No	209 (83.6)
Adjuvant endocrine therapy	—
Yes	170 (68.0)
No	80 (32.0)

^a^ Confirmed by BUS.

SLN, supraclavicular lymph nodes; NAC, neoadjuvant chemotherapy; SLND, supraclavicular lymph node dissection.

### 3.2 Surgical approach and survival outcomes

**FIGURE 1:**
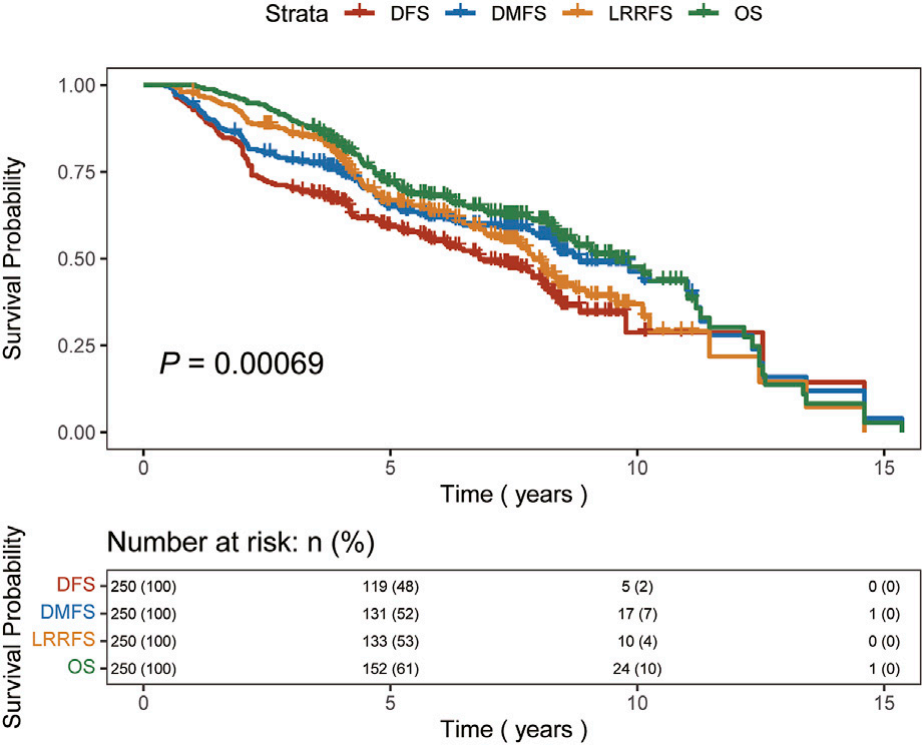
Kaplan-Meier curves of overall survival (OS), disease-free survival (DFS), locoregional recur­rence-free survival (LRRFS), and distant metastasis-free survival (DMFS) for breast cancer patients with ISLNM.

The median follow-up time among all patients in this study was 5.92 years, with a range of 1.05–15.36 years. The 5-year OS rate was 71.89%, while the DFS rate, LRRFS rate, and DMFS rates were 59.25%, 66.38%, and 64.98%, respectively. The 10-year OS rate, DFS rate, LRRFS rate, and DMFS rates were 45.71%, 28.76%, 33.26%, and 43.57%, respectively. The median OS time was 9.57 years and the median DFS time was 6.81 years, while the median LRRFS and DMFS were 7.91 years and 8.84 years, respectively. Figure 1 shows the Kaplan-Meier curves of OS, DFS, LRRFS, and DMFS for breast cancer patients with ISLNM.

Of the 250 patients included in the analysis, 185 (74.0%) underwent level I/II axillary dissection only, while 65 (26.0%) underwent aggressive locoregional surgical treatment (level I/II/III ± SLND), including 37 (14.8%) who underwent levels I/II/III (infraclavicular) dissection and 28 (11.2%) who underwent levels I/II/III with SLND. The clinicopathological characteristics of two subgroup are shown in Supplementary Tables S1, S2.

**FIGURE 2:**
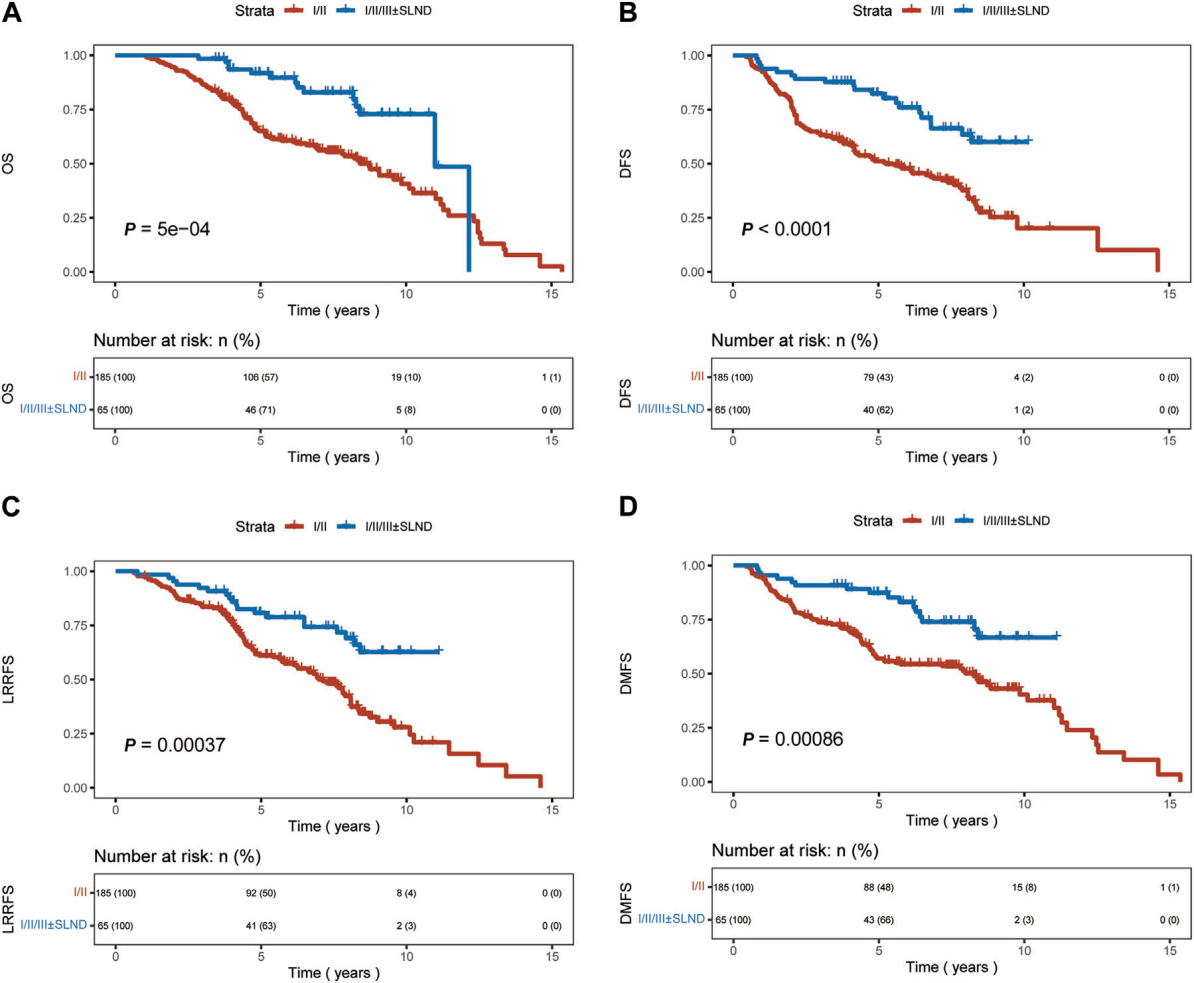
Kaplan-Meier curves of overall survival (OS), disease-free survival (DFS), locoregional recurrence-free survival (LRRFS), and distant metastasis-free survival (DMFS) for patients who underwent I/II axillary dissection only, and those with 1/11/lll ± SLND. **(A)** OS. **(B)** DFS. **(C)** LRRFS. **(D)** DMFS.

**FIGURE 3:**
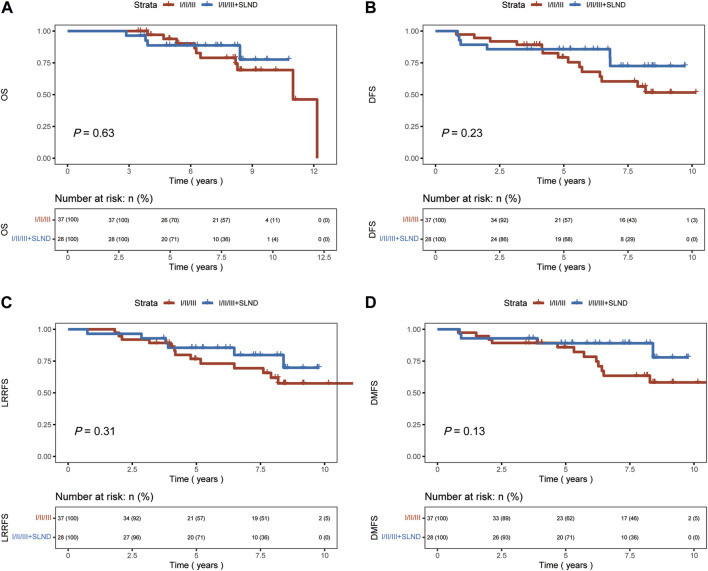
Kaplan-Meier curves of overall survival (OS), disease-free survival (DFS), locoregional recurrence-free survival (LRRFS), and distant metastasis-free survival (DMFS) for patients who underwent 1/11/111 dissection, and those with 1/11/lll + SLND. **(A)** OS. **(B)** DFS. **(C)** LRRFS. **(D)** DMFS.

A significant difference in OS, DFS, and DMFS was observed between patients who underwent levels I/II axillary dissection only and those who underwent aggressive locoregional surgery (levels I/II/III lymph node dissection ± SLND) (*p* < 0.01 Figure 2). The results indicated that the implementation of a comprehensive surgical strategy targeting the local and regional areas had a beneficial impact on the rates of recurrence, metastasis, and overall survival.Nevertheless, upon comparing the patient groups, specifically those who underwent levels I/II/III dissection and those who underwent levels I/II/III combined with SLND, no significant differences were observed in terms of OS, DFS, LRRFS, or DMFS outcomes (*p* = 0.63, *p* = 0.23, *p* = 0.31, *p* = 0.13, respectively, Figure 3). This implies that the incorporation of SLND into stages I/II/III dissection may not provide any further benefits in terms of survival.

### 3.3 Prognostic factors

Our study’s univariate analysis identified certain factors, including the nature of primary nodal surgery (Level I/II/III ± SLND), T stage (specifically T2 stage), and ER+/HER2-status, as being significantly related to OS, as detailed in Table 2 (*p* < 0.05). When delving into a multivariate logistic regression analysis that incorporated factors such as the primary nodal surgery type, breast surgery type, T stage, and receptor status (*p* < 0.15, Table 2), both the nodal surgery (Level I/II/III ± SLND) and T stage (T2) emerged as significantly linked to OS (*p* = 0.006 and *p* = 0.026, Table 2).

**TABLE 2:** Univariate and multivariate analysis using Cox-regression model of the survival of patients with ISLNM.

Variables	Overall survival				Disease-free survival			
	Univariate analysis		Multivariate analysis		Univariate analysis		Multivariate analysis	
	*P*	HR (95% CI)	*P*	HR (95% CI)	*P*	HR (95% CI)	*P*	HR (95% CI)
**Age, years**								
<50								
≥50	0.709	1.003 (0.986–1.021)			0.280	1.009 (0.993–1.024)		
**Pre-NAC largest SLN size** ^a^								
<1 cm								
≥1 cm	0.298	1.217 (0.841–1.760)			**0.025**	1.490 (1.052–2.111)	0.237	1.243 (0.867–1.782)
**Clinical T-stage**						-		
T1								
T2	**0.045**	1.729 (1.012–2.954)	**0.026**	1.895 (1.080–3.324)	0.239	1.348 (0.820–2.216)	0.352	1.275 (0.765–2.124)
T3	0.693	1.137 (0.600–2.155)	0.508	1.250 (0.645–2.425)	0.360	1.306 (0.738–2.310)	0.688	1.126 (0.631–2.008)
T4	0.070	1.783 (0.954–3.331)	0.064	1.861 (0.964–3.593)	0.057	1.709 (0.983–3.151)	0.234	1.440 (0.789–2.627)
**Histology**								
Invasive ductal carcinoma								
Others	0.439	0.575 (0.141–2.338)			0.368	0.526 (0.130–2.130)		
**Histologic grade**								
Low								
Median	0.385	0.808 (0.499–1.308)			0.525	1.154 (0.742–1.793)	0.590	1.149 (0.693–1.906)
High	0.516	1.201 (0.691–2.086)			**0.026**	1.790 (1.072–2.989)	**0.032**	1.859 (1.056–3.273)
**Receptor Status**								
ER+/HER2-								
ER+/HER2+	**0.028**	0.593 (0.372–0.946)	0.072	0.647 (0.403–1.039)	**<0.001**	0.432 (0.282–0.661)	**0.001**	0.459 (0.298–0.709)
ER-/HER2+	0.409	0.8082 (0.476–1.353)	0.835	1.064 (0.595–1.900)	**0.047**	0.604 (0.367–0.994)	0.270	0.270 (0.428–1.268)
ER-/HER2-	0.201	0.708 (0.417–1.202)	0.728	0.893 (0.472–1.689)	**0.045**	0.604 (0.369–0.989)	0.353	0.353 (0.403–1.384)
**Type of primary breast surgery**								
Yes								
No	0.097	0.377 (0.119–1.193)	0.89	1.103 (0.271–4.521)	0.074	0.402 (0.148–1.092)	0.362	0.594 (0.194–1.819)
**Type of primary nodal surgery**								
Level I/II								
Level I/II/III ± SLND	**0.001**	0.382 (0.217–0.671)	**0.006**	0.331 (0.152–0.722)	**<0.001**	0.379 (0.235–0.610)	**0.007**	0.417 (0.221–0.785)
**Tumor chemotherapy response (ypT0)**	0.81	1.05 (0.68–1.63)						
Yes								
No	0.913	0.976 (0.629–1.514)			0.988	0.997 (0.671–1.481)		
**Nodal pathologic complete response (ypN0)**								
Yes								
No	0.828	1.049 (0.682–1.613)			0.429	0.851 (0.570–1.269)		
**SLN radiographic response to NAC** ^a^								
Yes								
No	0.876	0.489 (0.603–1.274)			0.769	0.949 (0.671–1.343)		
**Post-NAC largest SLN size** ^a^								
<1 cm								
≥1 cm	0.533	1.204 (0.672–2.159)			0.841	1.509 (0.605–1.853)		
**Involvement of infraclavicular lymph nodes after NAC**								
Yes								
No	0.106	0.531 (0.246–1.145)	0.500	1.441 (0.498–4.167)	**0.023**	0.456 (0.232–0.898)	0.993	1.004 (0.412–2.446)
**SLN cumulative dose, Gy**								
<60								
≥60	0.299	1.259 (0.815–1.944)			0.563	1.127 (0.751–1.690)		
**Adjuvant targeted therapy**								
Yes								
No	0.100	0.649 (0.387–1.086)	0.098	0.579 (0.304–1.106)	0.228	0.742 (0.456–1.206)		
**Adjuvant endocrine therapy**								
Yes								
No	0.369	1.203 (0.804–1.800)			0.293	1.127 (0.840–1.782)		

^a^ Confirmed by BUS.

SLN, supraclavicular lymph nodes; NAC, neoadjuvant chemotherapy; SLND, supraclavicular lymph node dissection.

The bold value indicates a *p*-value less than 0.05.

Regarding DFS, the univariate analysis pinpointed factors, namely, the primary nodal surgery (Level I/II/III ± SLND), size of the largest supraclavicular lymph node prior to NAC, post-NAC infraclavicular lymph node involvement, histologic grade (specifically Grade 3), and receptor status, as being significantly associated with DFS (*p* < 0.05, Table 2). Upon performing a multivariate logistic regression analysis, which incorporated variables such as primary nodal surgery type, pre-NAC supraclavicular lymph node size, receptor status, post-NAC infraclavicular lymph node involvement, and primary breast surgery type (*p* < 0.15, Table 2), it was discerned that nodal surgery (Level I/II/III ± SLND), ER+/HER2-status, and histologic grade (Grade 3) stood out as independent determinants influencing DFS (*p* = 0.02, Table 2). These results provide insight into the factors that may impact survival outcomes in patients with ISLNM.

### 3.4 Adjuvant treatment

Before surgery, chemotherapy was administered to all patients in our study, averaging six rounds (ranging from one to eight cycles). A total of 156 patients, accounting for 62.4% of the sample, received postoperative chemotherapy. The average number of cycles administered was two, with a range of 1–8 cycles. Consistently, regimens based on anthracyclines and/or taxanes were employed. Within the cohort of patients, a total of 93 individuals, accounting for 37.2% of the sample, were identified as having HER2-positive disease based on the diagnostic methods of immunohistochemistry or fluorescence *in situ* hybridization. Out of these individuals, 41 patients, equivalent to 44.1% of the HER2-positive subgroup, received treatment specifically targeting the HER2 protein. No patients were prescribed preoperative endocrine treatment. However, a total of 170 patients, accounting for 68.0% of those diagnosed with ER/PR-positive breast cancer, received adjuvant endocrine therapy. Regarding the application of radiotherapy, a significant proportion of our study participants, specifically 91.2% (228 individuals), underwent this therapeutic intervention. This ensured that all patients got irradiation to the supraclavicular, infraclavicular, and axilla level II-III nodes, with the inclusion of the metastatic supraclavicular (SCV) node or its nodal bed boost, as deemed necessary. The primary radiation dose administered to the chest wall/breast and regional lymphatics was 50 Gy, with a range of 43.5–60 Gy, delivered over a course of 25 fractions, varying between 15 and 33 fractions, for 92.1% of the patients in this study. A small proportion, specifically 5.7%, was administered a radiation dosage of 43.5 Gy divided into 15 portions. The cumulative dose administered to metastatic SCV nodes or the nodal bed had an average of 60 Gy (with a range of 43.5–66 Gy) delivered over 28 fractions (encompassing 25–35) and 43.5 Gy (with a range of 43.5–52.2 Gy) delivered over 15 fractions (reaching 15–18). This distinction provides a clear explanation of the therapeutic techniques employed in our research.

### 3.5 Safety and toxicities

Among the 67 patients who underwent aggressive locoregional surgery, none experienced postoperative complications such as supraclavicular hemorrhage, edema, cervical chylous lymphocele, head and neck movement disorders, pleural effusion, or chylothorax. Postoperative over-drainage, defined as a 24-h volume of drainage greater than 200 mL, was noted in 48 patients (19.2%) and managed by compression and drainage without secondary surgery.

Radiotherapy side effects occurred in 63 patients, all of whom experienced grade 1–2 events of radiation dermatitis and 32 of whom also experienced grade 1–2 events of radiation esophagitis. No grade *≥* 3 acute or late toxicities were observed. These results suggest that the treatments used in this study were generally well-tolerated by patients.

## 4 Discussion

Breast cancer is the most common malignant tumor in females worldwide, with rapidly increasing incidence rates in China and other parts of Asia (Lei et al., 2021). However, compared to Western countries, where the detection rate of early breast cancer is over 60%, the detection rate in China is less than 30% (Duggan et al., 2021). As a result, the majority of breast cancer patients are diagnosed at the middle or late stages, with frequent metastasis to axillary and supraclavicular lymph nodes. Breast cancer with ISLNM is generally considered an indicator of poor prognosis, and the fifth edition of the AJCC TNM staging system defines it as M1 due to its poor outcome and the likelihood of developing distant metastasis within 1 year (Debois, 1997).

However, with improvements in diagnosis and multidisciplinary approaches, patient outcomes are improving (Brito et al., 2001) were the first to report that patients with breast cancer and ISLNM who received multimodal comprehensive treatment had a 5-year overall survival rate of 41.1%, significantly superior to that of patients with distant metastasis. Similar findings were subsequently reported by Chen et al. (2006), Huang et al. (2007), Fan et al. (2010), Park et al. (2011), Ogino et al. (2011), Dellapasqua et al. (2014), and Noh et al. (2015), which are comparable to those with stages IIIb/c disease but distinct from those with stage IV disease. As a result, the sixth edition of the AJCC-TNM breast cancer staging system reclassified breast cancer with ISLNM as stage IIIc instead of stage IV, as it is no longer considered to be distant metastasis (Singletary et al., 2002). Consequently, patients with ISLNM but no other evidence of distant metastases should receive therapeutic interventions aimed at achieving a curative outcome; i.e., combined-modality therapy consisting of NAC, targeted therapy, surgery, radiotherapy, and endocrine therapy.

In terms of surgery, there is still controversy regarding the treatment method for the local supraclavicular lymph node region. The latest NCCN guidelines recommend total or partial mastectomy with axillary dissection limited to axillary levels I and II for patients achieving clinical remission, with residual tumors in infraclavicular and supraclavicular regions usually managed through radiation (Gradishar et al., 2022). While the majority of studies indicate that locoregional control rates can exceed 80%, the 5-year OS and DFS rates typically remain at approximately 50%, ranging from 33.3% to 47% and 25%–34%, respectively (Brito et al., 2001; Huang et al., 2007; Park et al., 2011; Chang et al., 2013; Noh et al., 2015; Kim et al., 2020). This may be due to residual tumor leading to tumor development and growth because the dose of supraclavicular radiotherapy did not reach the radical dose, which is limited by surrounding vital organs and blood vessels such as the brachial plexus nerve. Moreover, complications from supraclavicular radiotherapy cannot be ignored. Radioactive nerve damage, especially brachial plexus injury induced by high-dose radiation therapy, may cause allodynia and movement disorder of the affected limb. Other common radiotherapy complications such as radiation dermatitis and radiation pneumonitis can reduce patients’ quality of life. In a study by the Texas MD Anderson Cancer Center, 156 patients reached a cumulative SCV dose ≥60 Gy and 120 of them experienced radiation dermatitis grade ≥2 while 13 patients experienced hyperpigmentation grade ≥2. Incidences of such events increase with dose and irradiated volume while radiation dose limits may lead to insufficient doses in treating tumors and failure of local control. Thus, radical excision of tumors in infraclavicular and supraclavicular regions may be a more thorough and effective treatment. Due to the special anatomical location of the SLN and the lack of a unified standard for the extent of lymph node dissection, studies on aggressive locoregional surgery for ISLNM have been unsystematic and sporadic, with no clear recommendation in the NCCN guidelines. The primary question is whether more radical surgery can improve local control, reduce distant metastasis, and prolong survival outcomes for breast cancer patients with ISLNM.

A review of current literature between January 1975 and June 2020 using PubMed and Web of Science databases found 115 studies on breast cancer with ISLNM, but only 10 studies on the role of SLND. The largest study to date, by Ai et al. (2020), included 146 patients who received SLND with RT and 159 patients who received RT alone. They found that SLND with RT was not associated with superior survival overall, but in stratified analyses, patients with non-luminal A tumors and 4-9 positive axillary lymph nodes who underwent SLND with RT had superior OS and DFS compared to those who received RT alone. Jung et al. (2015) also found that the 5-year OS for 73 patients who received local aggressive treatment (SLND + RT) was superior to that of 38 patients who received non-aggressive treatment. However, other studies have reported opposite conclusions. Diao et al. (2022) found that SLND had a limited role, as evidenced by comparable LRRFS rates with and without neck dissection. This conclusion is supported by Kim et al., who reported no improvement in locoregional control or DFS with SLND. Similar trends were observed by Ma et al. (2020), Chang et al. (2013), and Sun et al. (2020). In summary, the available evidence on the role of SLND in the treatment of breast cancer with ISLNM is mixed and further research is needed to determine its effectiveness in improving patient outcomes. The optimal extent of SLND is a topic of debate. The supraclavicular fossa is defined as the IV region, upper part of the V region of the neck, and the entire supraclavicular region. Clinically, SLND involves removing lymph nodes containing adipose tissue in the triangle formed by the posterior border of the sternocleidomastoid muscle, the inferior edge of the omohyoid muscle, and the upper border of the clavicle. The lymph nodes of regions II and III are not reliably visualized as conventional lymphatic drainage from the breasts and are staged as M1 (Sesterhenn et al., 2006). After reviewing the published literature (Table 3), we made a forest plot to summarize the available data (Supplementary Figure S1) and conducted a retrospective study at our center. Our results show a marked increase in 5-year OS and DFS rates to 71.89% and 59.25%, respectively, when compared to historical outcomes. This improvement in long-term survival may be attributed to the implementation of more aggressive locoregional management strategies, such as infraclavicular lymph node dissection and SLND.

**TABLE 3:** Summary of studies on SLND for breast cancer patients with ISLNM.

Author, year	Study period	No.	No. SLND	Follow-up (years)	5-yr OS (%)	5-yr DFS/PFS/RFS (%)	*P* of SLND + RT VS. RT
Chang (2013)	2005.1–2005.10	29	13	3.92	46.2%	NA	NS
June 2015	1990–2010	111	73	NA	64.2%	56.2%	S
Diao (2022)	2004–2016	173	10	2.83	73%	50%	NS
Kim (2020)	2000–2014	78	35	4.65	68.6%	68.4%	NS
Ma (2020)	2009–2019	99	27	3.00	76.8% (3-yr)	41.4%	NS
Sun (2020)	2010–2019	108	84	6.25	67.8%	30.6%	NS
Ai (2020)	2004–2017	305	146	4.00	73.9%	54.8%	NS; S in stratified analyses
Ai (2020)	2018.1–2018.12	61	25	-	-	-	NS
Liu (2021)	2000–2016	142	104	NA	NA	NA	S for RFS NS for OS and DMFS

SLND, supraclavicular lymph node dissection; ISLNM, ipsilateral supraclavicular lymph node metastasis; OS, overall survival; DFS, disease-free survival; DMFS, distant metastasis-free survival; NS, no significant; S, significant; NA, not available.

In our study, we categorized axillary surgery into two groups: levels I/II axillary dissection only and aggressive locoregional surgery group including levels I/II/III ± SLND. The results indicate that patients who underwent aggressive locoregional surgery experienced significant benefits in OS, DFS, LRRFS, and DMFS (*p* < 0.01). Further analysis comparing the effects of two types of aggressive locoregional surgery on survival outcomes indicated that the addition of SLND to levels I/II/III surgery did not confer a significant advantage in terms of OS, DFS, LRRFS, or DMFS.

Moreover, the implementation of ultrasound-guided sentinel lymph node biopsy has facilitated precise staging and treatment planning for patients. Our institution achieved a notable proportion of patients with biopsy tissue-confirmed SLN involvement (99.6%). Reasons for excluding biopsy tissue test results typically included initial assessment at an external facility, commencement of systemic therapy prior to imaging or diagnosis of sentinel lymph node disease, presence of imaging adequate for diagnosis (usually involving large, positively identified nodes through positron emission tomography), or anatomically unfavorable location of the node necessitating biopsy tissue testing on a more accessible node.

A significant limitation of existing studies on breast cancer with ISLNM is their failure to specifically examine LRRFS. For example, Kim et al. (2020) conducted a study involving 78 patients with ipsilateral cervical lymph node involvement at presentation and reported a 5-year LRRFS rate of 68%, which is comparable to our findings. However, the low 5-year LRRFS rate may be attributed to the fact that only 83% of patients received radiation fields encompassing the ipsilateral cervical lymph nodes, and only 3% underwent SLND. Our study reports a 5-year LRRFS rate of 66.38%, which may have been influenced by the fact that only 65 (26.0%) patients underwent aggressive locoregional surgery. In order to determine any relationship between clinical features, treatment, and survival, univariate and multivariate analyses of prognostic factors were carried out. In both the univariate and multivariate analyses, nodal surgery as level I/II/III ± SLND surgery was significantly associated with OS and DFS. This result indicates that aggressive locoregional surgery may be a strong prognostic factor.

RT after surgery for breast cancer with ISLNM has provided acceptable in-field regional control rates in previous studies. In regards to radiotherapy dose, trials have established the role of adjuvant breast RT after a lumpectomy using a dose of 50 Gy in 25 fractions (Veronesi et al., 1981; Poortmans et al., 2015), and 50 Gy for elective regional nodal radiation (Poortmans et al., 2015). However, the optimal dose for gross disease and high-risk nodal regions in cN3c-stage breast cancer is unknown. The radiotherapy dose presented in the published literature was mainly 50 Gy, with the fields of radiotherapy chest wall generally including axilla, infraclavicular, and supraclavicular areas. Some clinicians have suggested a dose of 46–50 Gy in 23–25 fractions to the breast/chest wall and regional nodes without an SLN boost, while others have prescribed 50.4 Gy in 28 fractions with an SLN boost ≥54 Gy. Diao et al. (2022) investigated the optimal dose for patients with ISLNM as 156 patients (90%) received a cumulative SLN dose of ≥60 Gy. They found that a cumulative SLN dose of ≥60 Gy was associated with improved OS but not LRRFS or DFS. In our study, 56 patients (22.4%) had an SLN boost and the cumulative dose was ≥60 Gy, and RT was well-tolerated as no grade ≥3 acute or late toxicities were observed.

This study has certain limitations due to its retrospective design. Specifically, the retrospective nature of data collection limits the strength of statistical comparisons and the inferences that can be made. Additionally, the non-randomized retrospective nature of the study precludes random assignment of treatment types, which were instead selected individually based on the patient’s condition. This selection of treatment may have been biased, limiting the interpretation of the survival analysis. The study interval was extensive, and the emergence of systemic treatment resulted in some clinical data being absent. While a preliminary evaluation suggested that the enhancement of treatment modalities had a negligible effect on the survival analysis, it may still introduce some degree of deviation. Further large-scale, randomized studies are necessary to evaluate the benefits of neck surgery in patients with ISLNM.

Despite these limitations, this study has several strengths. It is one of the largest examinations of individuals with N3c breast cancer, with a median follow-up duration (5.92 years) that exceeds that of previously reported literature. The cohort was divided into three distinct groups based on the extent of axillary surgery: level I/II axillary dissection, levels I/II/III (infraclavicular) dissection, and level I/II/III + SLND group. Additionally, published literature on this topic was reviewed and summarized in a forest plot. The study aimed to investigate the influence of the extent of surgery on survival outcomes. While more comprehensive stratification requires a larger sample size to accurately identify patients who may benefit from SLND, this study provides valuable insights and directions for future research.

## 5 Conclusion

Breast cancer with ISLNM may be classified as a locoregional disease that requires a combination of systemic and local therapies. The incorporation of more aggressive regional therapies, including level I/II/III ± SLND surgery, as part of a multimodal treatment strategy, may yield favorable outcomes in terms of recurrence, metastasis, and survival. This approach may give patients with ISLNM the best chance for a positive outcome.

## Data Availability

The original contributions presented in the study are included in the article/Supplementary Material, further inquiries can be directed to the corresponding authors.
