# Supplementary material for: Role of aggressive locoregional surgery in treatment strategies for ipsilateral supraclavicular lymph node metastasis of breast cancer: a real-world cohort study
Source: Front Mol Biosci. 2023 Oct 17;10:1248410. doi: 10.3389/fmolb.2023.1248410 (PMC10616842; doi:10.3389/fmolb.2023.1248410)
Supplement: Supplementary file 1 [file DataSheet1.docx]

Supplementary Material

Role of aggressive locoregional surgery in treatment strategies for ipsilateral supraclavicular lymph node metastasis of breast cancer: a real-world cohort study

**Kexin Feng^1†^, Zeyu Xing^1†^, Qichen Dai^1^, Han Cheng^1^, Yu Tang^2^*, Xiang Wang^1^***

† These authors contributed equally to this work and share first authorship

1. Department of Breast Surgical Oncology, National Cancer Center/National Clinical Research Center for Cancer/Cancer Hospital, Chinese Academy of Medical Sciences and Peking Union Medical College, Beijing, 100021, China
2. GCP center, National Cancer Center/National Clinical Research Center for Cancer/Cancer Hospital, Chinese Academy of Medical Sciences and Peking Union Medical College, Beijing 100021, China

*** Correspondence:**

Yu Tang, [tangyu@cicams.an.cn](mailto:tangyu@cicams.an.cn)

Xiang Wang, xiangw@vip.sina.com

Keywords: Breast cancer; supraclavicular lymph node; supraclavicular lymph node dissection (SLND); survival; prognostic factors

# Supplementary Figures and Tables

## Supplementary Figures


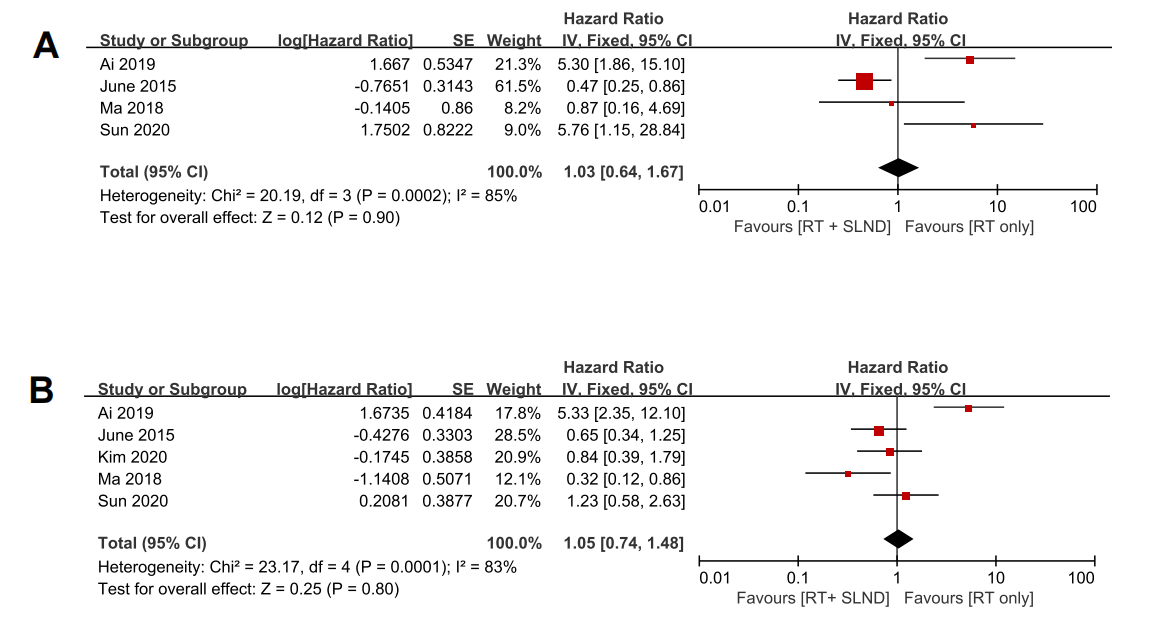


**Supplementary Figure 1.** Forest plot of hazard ratio (HR) of supraclavicular lymph node dissection (SLND) plus radiotherapy (RT) for overall survival (OS) and disease-free survival (DFS). (A) Forest plot of HR of SLND + RT for OS. (B) Forest plot of HR of SLND + RT for DFS.

## 1.2 Supplementary Tables

**Table S1.** **Patient and treatment characteristics of patients with level I/II axillary dissection and level I/II/III±SLND surgery.**

| **Variable** | **I/II**  **n=185** | **I/II/III±SLND**  **n=65** | ***P*** |
| --- | --- | --- | --- |
| Age, median | 52.00[43.00;59.00] | 48.00[39.50,54.50] | 0.018 |
| Pre-NAC largest SLN size^*^ |  |  | 0.011 |
| ＜ 1cm | 77(41.6) | 39(60.0) |  |
| ≥ 1cm | 108(58.4) | 26(40.0) |  |
| Clinical T-stage |  |  | 0.499 |
| T1 | 31(16.8) | 16(24.6) |  |
| T2 | 86(46.5) | 30(46.2) |  |
| T3 | 40(21.6) | 11(16.9) |  |
| T4 | 28(15.1) | 8(12.3) |  |
| Histologic grade |  |  | 0.302 |
| Invasive ductal carcinoma | 182(98.4) | 65(100.0) |  |
| Others | 3(1.6) | 0(0.0) |  |
| Histologic grade |  |  | 0.816 |
| Low | 43(23.2) | 17(26.2) |  |
| Median | 105(56.8) | 37(56.9) |  |
| High | 37(20.0) | 11(16.9) |  |
| Receptor Status |  |  | 0.128 |
| ER+/HER2- | 67(36.2) | 29(44.6) |  |
| ER+/HER2+ | 30(16.2) | 15(23.1) |  |
| ER-/HER2+* | 41(22.2) | 7(10.8) |  |
| ER-/HER2- | 47(25.4) | 14(21.5) |  |
| Type of primary breast surgery |  |  | 0.232 |
| Yes | 181(97.8) | 65(100.0) |  |
| No | 4(2.2) | 0(0.0) |  |
| Tumor chemotherapy response (ypT0) |  |  | 0.149 |
| Yes | 42(23.2) | 21(32.3) |  |
| No | 139(76.8) | 44(67.7) |  |
| Nodal pathologic complete response (ypN0) |  |  | 0.754 |
| Yes | 51(28.2) | 17(26.2) |  |
| No | 130(71.8) | 48(73.8) |  |
| SLN radiographic response to NAC^*^ |  |  | 0.769 |
| Yes | 110(59.5) | 40(61.5) |  |
| No | 75(40.5) | 25(38.5) |  |
| Involvement of infraclavicular lymph nodes after NAC |  |  | <0.001 |
| Yes | 0(0.0) | 26(40.0) |  |
| No | 185(100.0) | 39(60.0) |  |
| Post-NAC largest SLN size^*^ |  |  | 0.192 |
| ＜ 1cm | 163(88.1) | 61(93.8) |  |
| ≥ 1cm | 22(11.9) | 4(6.2) |  |
| SLN cumulative dose, Gy |  |  | <0.001 |
| ＜ 60 | 107(65.6) | 65(100.0) |  |
| ≥ 60 | 56(34.4) | 0(0.0) |  |
| Adjuvant HER-2 targeted therapy |  |  | 0.518 |
| Yes | 32(17.3) | 9(13.8) |  |
| No | 153(82.7) | 56(86.2) |  |
| Adjuvant endocrine therapy |  |  | 0.108 |
| Yes | 131(70.8) | 39(60.0) |  |
| No | 54(29.2) | 26(40.0) |  |

^*^Confirmed by BUS

SLN, supraclavicular lymph nodes; NAC, neoadjuvant chemotherapy; SLND, supraclavicular lymph node dissection

**Table S2. Patient and treatment characteristics of patients with level I/II/III dissection and level I/II/III+SLND surgery.**

| **Variable** | **I/II/III**  **n=37** | **I/II/III+SLND**  **n=28** | ***P*** |
| --- | --- | --- | --- |
| Age, median [IQR] | 52.00[43.00;59.00] | 48.00[39.50,54.50] | 0.624 |
| Pre-NAC largest SLN size^*^ |  |  | 0.368 |
| ＜ 1cm | 20(54.1) | 19(67.9) |  |
| ≥ 1cm | 17(45.9) | 9(32.1) |  |
| Clinical T-stage |  |  | 0.948 |
| T1 | 9(24.3) | 7(25.0) |  |
| T2 | 17(45.9) | 13(46.4) |  |
| T3 | 7(18.9) | 4(14.3) |  |
| T4 | 4(10.8) | 4(14.3) |  |
| Histologic grade |  |  | - |
| Invasive ductal carcinoma | 37(100.0) | 28(100.0) |  |
| Others | 0(0.0) | 0(0.0) |  |
| Histologic grade |  |  | 0.752 |
| Low | 11(29.7) | 6(21.4) |  |
| Median | 20(54.1) | 17(60.7) |  |
| High | 6(16.2) | 5(17.9) |  |
| Receptor Status |  |  | 0.881 |
| ER+/HER2- | 18(48.6) | 11(39.3) |  |
| ER+/HER2+ | 8(21.6) | 7(25.0) |  |
| ER-/HER2+ | 4(10.8) | 3(10.7) |  |
| ER-/HER2- | 7(18.9) | 7(25.0) |  |
| Type of primary breast surgery |  |  | 0.348 |
| Mastectomy | 32(86.5) | 27(96.4) |  |
| Lumpectomy | 5(13.5) | 1(3.6) |  |
| Tumor chemotherapy response (ypT0) |  |  | 0.980 |
| Yes | 12(32.4) | 9(32.1) |  |
| No | 25(67.6) | 19(67.9) |  |
| Nodal pathologic complete response (ypN0) |  |  | 0.451 |
| Yes | 11(29.7) | 6(21.4) |  |
| No | 26(70.3) | 22(78.6) |  |
| SLN radiographic response to NAC^*^ |  |  | 0.362 |
| Yes | 16(43.2) | 9(32.1) |  |
| No | 21(56.8) | 19(67.9) |  |
| Involvement of infraclavicular lymph nodes after NAC |  |  | <0.001 |
| Yes | 11(29.7) | 28(100.0) |  |
| No | 26(70.3) | 0(0.0) |  |
| Post-NAC largest SLN size^*^ |  |  | 0.458 |
| ＜ 1cm | 33(89.2) | 28(100.0) |  |
| ≥ 1cm | 4(10.8) | 0(0.0) |  |
| SLN cumulative dose, Gy |  |  | - |
| ＜ 60 | 37(100.0) | 28(100.0) |  |
| ≥ 60 | 0(0.0) | 0(0.0) |  |
| Adjuvant HER-2 targeted therapy |  |  | 0.057 |
| Yes | 35(94.6) | 21(75.0) |  |
| No | 2(5.4) | 7(25.0) |  |
| Adjuvant endocrine therapy |  |  | 0.357 |
| Yes | 13(35.1) | 13(46.4) |  |
| No | 24(64.9) | 15(53.6) |  |

^*^Confirmed by BUS

SLN, supraclavicular lymph nodes; NAC, neoadjuvant chemotherapy; SLND, supraclavicular lymph node dissection
